# Supplementary material for: Causal Relationship between Aging and Anorexia Nervosa: A White-Matter-Microstructure-Mediated Mendelian Randomization Analysis
Source: Biomedicines. 2024 Aug 16;12(8):1874. doi: 10.3390/biomedicines12081874 (PMC11351342; doi:10.3390/biomedicines12081874)
Supplement: Supplementary file 1 [file biomedicines-12-01874-s001.zip › Supplementary Figure/Figure S4/Singe SNP plot of MR analysis for TL to ubm-b-1749.pdf]

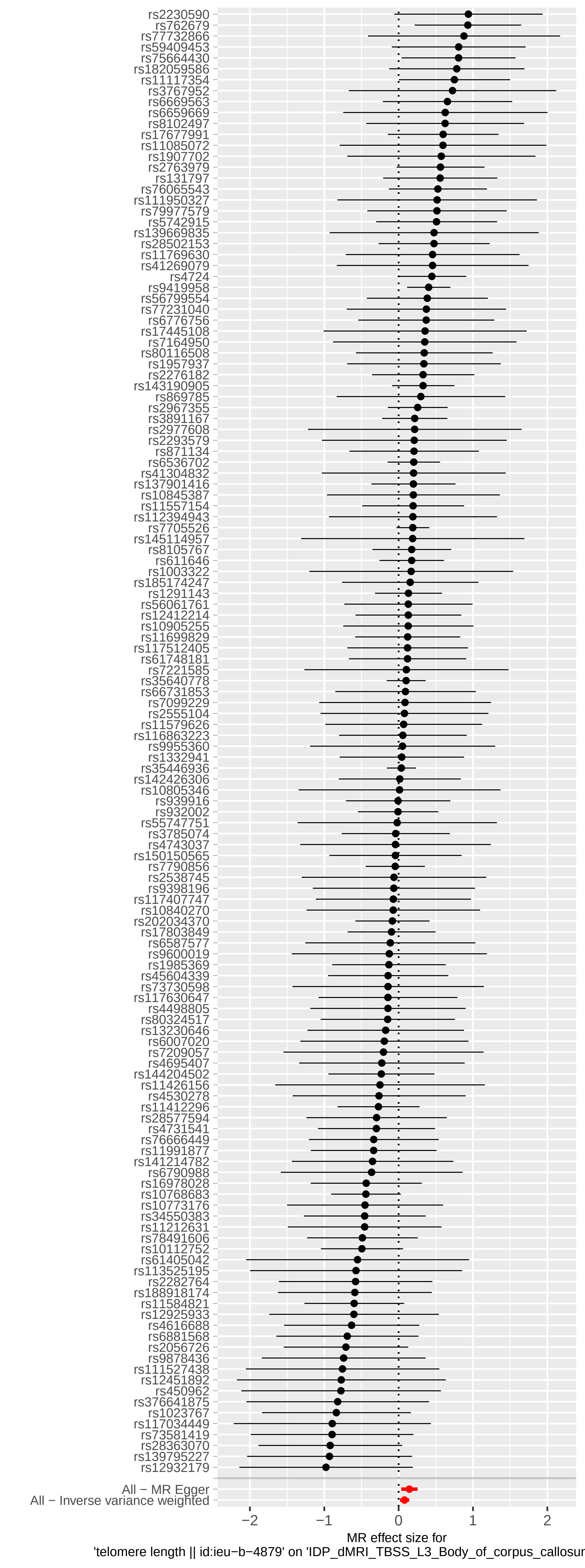
rs2230590
rs762679
rs77732866
rs59409453
rs75664430
rs182059586
rs11117354
rs3767952
rs6669563
rs6659669
rs8102497
rs17677991
rs11085072
rs1907702
rs2763979
rs131797
rs76065543
rs111950327
rs79977579
rs5742915
rs139669835
rs28502153
rs11769630
rs41269079
rs4724
rs9419958
rs56799554
rs77231040
rs6776756
rs17445108
rs7164950
rs80116508
rs1957937
rs2276182
rs143190905
rs869785
rs2967355
rs3891167
rs2977608
rs2293579
rs871134
rs6536702
rs41304832
rs137901416
rs10845387
rs11557154
rs112394943
rs7705526
rs145114957
rs8105767
rs611646
rs1003322
rs185174247
rs1291143
rs56061761
rs12412214
rs10905255
rs11699829
rs117512405
rs61748181
rs7221585
rs35640778
rs66731853
rs7099229
rs2555104
rs11579626
rs116863223
rs9955360
rs1332941
rs35446936
rs142426306
rs10805346
rs939916
rs932002
rs55747751
rs3785074
rs4743037
rs150150565
rs7790856
rs2538745
rs9398196
rs117407747
rs10840270
rs202034370
rs17803849
rs6587577
rs9600019
rs1985369
rs45604339
rs73730598
rs117630647
rs4498805
rs80324517
rs13230646
rs6007020
rs7209057
rs4695407
rs144204502
rs11426156
rs4530278
rs11412296
rs28577594
rs4731541
rs76666449
rs11991877
rs141214782
rs6790988
rs16978028
rs10768683
rs10773176
rs34550383
rs11212631
rs78491606
rs10112752
rs61405042
rs113525195
rs2282764
rs188918174
rs11584821
rs12925933
rs4616688
rs6881568
rs2056726
rs9878436
rs111527438
rs12451892
rs450962
rs376641875
rs1023767
rs117034449
rs73581419
rs28363070
rs139795227
rs12932179
All − MR Egger
All − Inverse variance weighted
−2
−1
0
1
2
MR effect size for
'telomere length || id:ieu−b−4879' on 'IDP_dMRI_TBSS_L3_Body_of_corpus_callosur
